# Common threads: epigenetics, metabolism and the clock

**DOI:** 10.1186/1471-2164-15-S2-O9

**Published:** 2014-04-02

**Authors:** Paolo Sassone-Corsi

**Affiliations:** 1 Center for Epigenetics and Metabolism, University of California, Irvine, California 92697, USA

## 

Circadian rhythms govern a number of fundamental physiological functions in almost all organisms, from prokaryotes to humans. The circadian clocks are intrinsic time-tracking systems with which organisms can anticipate environmental changes and adapt to the appropriate time of day. Disruption of these rhythms can have a profound influence to human health and has been linked to depression, insomnia, jet lag, coronary heart disease, neurodegenerative disorders and cancer. At the heart of circadian regulatory pathways is the clock machinery, a remarkably coordinated transcription-translation system that utilizes also dynamic changes in chromatin transitions and epigenetic control. Recent findings indicate that regulation goes also the other way, since specific elements of the clock are able to sense changes in the cellular metabolism. Understanding in full detail the intimate links between cellular metabolism and the circadian clock machinery will provide not only critical insights into system physiology and endocrinology, but also novel avenues for pharmacological intervention towards metabolic disorders.

